# Supplementary material for: Long- versus short-duration systemic corticosteroid regimens for acute exacerbations of COPD: A systematic review and meta-analysis of randomized trials and cohort studies
Source: PLoS One. 2023 Dec 29;18(12):e0296470. doi: 10.1371/journal.pone.0296470 (PMC10756550; doi:10.1371/journal.pone.0296470)
Supplement: S1 Table — (PDF) [file pone.0296470.s003.pdf]

S1 Table. List of excluded studies and reasons for exclusion.

Study Name	Reason for exclusion
Abroug 2014	Compared prednisone with placebo
Burge 2003	Investigated the effect of prednisolone on subsequent treatment with bronchodilators without any comparison of durations
CORTICO-COP	Comparison between normal therapy and eosinophil-guided therapy
Davies 1999	Compared prednisolone with placebo
George 2020	Assessed hospital adherence to treatment guidelines
Johannesmeyer 2021	Compared corticosteroid dosage to hyperglycemia risk, but no comparison with duration
Karlsson 2017	Investigated the effect of prednisolone on exercise tolerance in men with stable COPD
Long 2018	A short summary of the 2018 Cochrane review
Ma 2021	Compared inhaled vs systemic corticosteroids
Magovern 2019	Brief summary of current knowledge regarding optimal corticosteroid regimen duration
Marcos 2017	Assessed hospital adherence to treatment guidelines
Matte 2018	Assessed the effect of a physician education session on adherence to recommended COPD corticosteroid treatment guidelines
Niewoehner 1999	Wrong durations; compared 2 weeks with 8 weeks and placebo
RECUT	Trial not complete, and wrong durations (3 vs 5 days)
Scheutz 2015	Post-hoc analysis of Leuppi 2013 data
Sivapalan 2021	Post-hoc analysis of two different trials
Willaert 2002	Compared different administration methods
